# Incidence of DAA failure and the clinical impact of retreatment in real-life patients treated in the advanced stage of liver disease: Interim evaluations from the PITER network

**DOI:** 10.1371/journal.pone.0185728

**Published:** 2017-10-04

**Authors:** Loreta A. Kondili, Giovanni Battista Gaeta, Maurizia Rossana Brunetto, Alfredo Di Leo, Andrea Iannone, Teresa Antonia Santantonio, Adele Giammario, Giovanni Raimondo, Roberto Filomia, Carmine Coppola, Daniela Caterina Amoruso, Pierluigi Blanc, Barbara Del Pin, Liliana Chemello, Luisa Cavalletto, Filomena Morisco, Laura Donnarumma, Maria Grazia Rumi, Antonio Gasbarrini, Massimo Siciliano, Marco Massari, Romina Corsini, Barbara Coco, Salvatore Madonia, Marco Cannizzaro, Anna Linda Zignego, Monica Monti, Francesco Paolo Russo, Alberto Zanetto, Marcello Persico, Mario Masarone, Erica Villa, Veronica Bernabucci, Gloria Taliani, Elisa Biliotti, Luchino Chessa, Maria Cristina Pasetto, Pietro Andreone, Marzia Margotti, Giuseppina Brancaccio, Donatella Ieluzzi, Guglielmo Borgia, Emanuela Zappulo, Vincenza Calvaruso, Salvatore Petta, Loredana Falzano, Maria Giovanna Quaranta, Liliana Elena Weimer, Stefano Rosato, Stefano Vella, Edoardo Giovanni Giannini

**Affiliations:** 1 Istituto Superiore di Sanità, Rome, Italy; 2 Second University of Naples, Naples, Italy; 3 University Hospital of Pisa, Pisa, Italy; 4 University of Pisa, Pisa, Italy; 5 University of Bari, Bari, Italy; 6 University of Foggia, Foggia, Italy; 7 University Hospital of Messina, Messina, Italy; 8 Gragnano Hospital, Gragnano, Italy; 9 S.M. Annunziata Hospital, Florence, Italy; 10 University Hospital of Padua, Padua, Italy; 11 University of Naples Federico II, Naples, Italy; 12 University of Milan, Milan, Italy; 13 Catholic University of Rome, Rome, Italy; 14 Arcispedale S. Maria Nuova-IRCCS, Reggio Emilia, Italy; 15 Villa-Sofia Cervello Hospital, Palermo, Italy; 16 University of Florence, Florence, Italy; 17 University of Padua, Padua, Italy; 18 University of Salerno, Salerno, Italy; 19 University of Modena and Reggio Emilia, Modena, Italy; 20 Sapienza University of Rome, Rome, Italy; 21 University of Cagliari, Cagliari, Italy; 22 University of Bologna, Bologna, Italy; 23 University Hospital of Verona, Verona, Italy; 24 University of Palermo, Palermo, Italy; 25 University of Genova, Genova, Italy; Kaohsiung Medical University Chung Ho Memorial Hospital, TAIWAN

## Abstract

**Background:**

Few data are available on the virological and clinical outcomes of advanced liver disease patients retreated after first-line DAA failure.

**Aim:**

To evaluate DAA failure incidence and the retreatment clinical impact in patients treated in the advanced liver disease stage.

**Methods:**

Data on HCV genotype, liver disease severity, and first and second line DAA regimens were prospectively collected in consecutive patients who reached the 12-week post-treatment and retreatment evaluations from January 2015 to December 2016 in 23 of the PITER network centers.

**Results:**

Among 3,830 patients with advanced fibrosis (F3) or cirrhosis, 139 (3.6%) failed to achieve SVR. Genotype 3, bilirubin levels >1.5mg/dl, platelet count <120,000/mm^3^ and the sofosbuvir+ribavirin regimen were independent predictors of failure by logistic regression analysis. The failure rate was 7.6% for patients treated with regimens that are no longer recommended or considered suboptimal (sofosbuvir+ribavirin or simeprevir+sofosbuvir±ribavirin), whereas 1.4% for regimens containing sofosbuvir combined with daclatasvir or ledipasvir or other DAAs. Of the patients who failed to achieve SVR, 72 (51.8%) were retreated with a second DAA regimen, specifically 38 (52.7%) with sofosbuvir+daclatasvir, 27 (37.5%) with sofosbuvir+ledipasvir, and 7 (9.7%) with other DAAs ±ribavirin. Among these, 69 (96%) patients achieved SVR12 and 3 (4%) failed. During a median time of 6 months (range: 5–14 months) between failure and the second DAA therapy, the Child-Pugh class worsened in 12 (16.7%) patients: from A to B in 10 patients (19.6%) and from B to C in 2 patients (10.5%), whereas it did not change in the remaining 60 patients. Following the retreatment SVR12 (median time of 6 months; range: 3–12 months), the Child-Pugh class improved in 17 (23.6%) patients: from B to A in 14 (19.4%) patients, from C to A in 1 patient (1.4%) and from C to B in 2 (2.9%) patients; it remained unchanged in 53 patients (73.6%) and worsened in 2 (2.8%) patients. Of patients who were retreated, 3 (4%) had undergone OLT before retreatment (all reached SVR12 following retreatment) and 2 (2.8%) underwent OLT after having achieved retreatment SVR12. Two (70%) of the 3 patients who failed to achieve SVR12 after retreatment, and 2 (2.8%) of the 69 patients who achieved retreatment SVR12 died from liver failure (Child-Pugh class deteriorated from B to C) or HCC complications.

**Conclusions:**

Failure rate following the first DAA regimen in patients with advanced disease is similar to or lower than that reported in clinical trials, although the majority of patients were treated with suboptimal regimens. Interim findings showed that worsening of liver function after failure, in terms of Child Pugh class deterioration, was improved by successful retreatment in about one third of retreated patients within a short follow-up period; however, in some advanced liver disease patients, clinical outcomes (Child Pugh class, HCC development, liver failure and death) were independent of viral eradication.

## Introduction

An estimated 71 million people worldwide are chronically infected with the hepatitis C virus (HCV) [1]. Chronic HCV infection places a large burden on health systems because it is one of the leading causes of cirrhosis, hepatocellular carcinoma (HCC) and orthotropic liver transplantation (OLT) [2,3]. Following the introduction of therapy with interferon (IFN)-free Direct Acting Antivirals (DAAs), the World Health Organization (WHO) set the goal of reducing liver-related deaths by 65% by the year 2030 [4].

For HCV therapy to be considered successful, its use should result in viral clearance with a sustained virologic response (SVR), as well as improved clinical outcomes. Following IFN-based treatment, SVR has been found to be associated with improved overall clinical outcomes [5,6]. In patients with compensated HCV-related cirrhosis, SVR was associated with slower disease progression, fewer liver-related mortalities, and the survival rate is similar to that of the general population [7–9]. Marked improvements in clinical outcomes following SVR have also been reported in patients with severe liver damage [10–12].

The universal treatment of all HCV-infected individuals has been hampered by the high cost of DAAs, which makes difficult for the payers to sustain their use. From January 2015 to March 2017, the Italian Medicines Agency limited the use of DAA to patients with advanced and progressive liver disease and to a few other categories of patients [13].

DAAs are highly effective, well tolerated and can be administered even in patients with advanced liver disease. However, the failure to eradicate HCV RNA with DAAs remains an important challenge mainly in this subset of patients [14]. The objective of the present analysis was to estimate the incidence of DAA failure and the clinical impact of retreatment in a real-life cohort of patients with advanced fibrosis/cirrhosis.

## Patients and methods

### Patients

The study was prospective in design and was conducted among patients attending 23 clinical centers involved in PITER (Italian Platform for the Study of Therapies for Viral Hepatitis) [15]. For the purpose of the present study, we retrieved all consecutive patients treated with DAA who reached the 12-week post-treatment HCV RNA evaluation from January 2015 to June 2016. The study population included patients eligible for DAA according to the indications of the Italian Medicines Agency, which limit use mainly to patients with advanced and progressive liver disease (i.e., fibrosis stage of F3 or higher), patients with severe extrahepatic HCV manifestations and patients who had undergone orthotropic liver transplantation (OLT), independently of the liver disease stage [13–16].

The DAAs available in Italy from January to May 2015 were sofosbuvir (SOF) and simeprevir (SIM), whereas since June 2015, daclatasvir and the fixed dose combinations ledipasvir (LDV)/SOF and ombitasvir/paritaprevir /ritonavir+dasabuvir (3D regimen) have also been available.

Data were collected on the HCV genotype, the liver disease stage and the DAA regimen used. Patients enrolled in clinical trials were excluded from the study. SVR was defined as being HCV-RNA-negative at the end of treatment and at the 12-week post-treatment evaluation. SVR was defined as undetectable HCV-RNA level as assessed by highly sensitive molecular methods (lower limit of detection ranging from < = 12 to < = 15 IU/ml), 12 weeks after completion of anti HCV therapy. This is referred to as the SVR12 and achieving an SVR12 is the goal of therapy. The fibrosis stage was defined based on liver transient elastography data, which were considered as validated if each patient had at least 10 valid stiffness measurements, with a success rate of at least 80%, an interquartile range of less than 30% of the median stiffness score, and a Body Mass Index of <30kg/m^2^. F3 fibrosis stage was classified if the stiffness score was within the range of 9.5–12.5 kPa. Liver disease was classified as “progressive/severe liver disease” if the stiffness score was equal to or higher than 12.5 kPa or if there were signs of liver cirrhosis (signs of portal hypertension) [17,18]. The diagnosis of cirrhosis was based on liver biopsy data or either transient elastography or a combination of clinical, laboratory and imaging findings.

The overall duration of the observation included the period from January 2015 to December 2016 (end of the period for SVR12 evaluation for patients who were retreated with a second DAA regimen during the period from January 2015 to June 2016). The following clinical data were collected at baseline following the first and second treatment and at the end of the follow-up of the second treatment: liver fibrosis stage and/or presence of clinical cirrhosis, Child-Pugh score and complications of severe liver disease, if present (i.e., ascites, encephalopathy, spontaneous bacterial peritonitis, esophageal varices, bleeding from esophageal varices, and HCC development). Patients underwent semiannual US surveillance, and the diagnosis of HCC was based on international guidelines [19]. Data were also collected on OLT and previous IFN treatment.

### Statistical analysis

Differences among the proportions were evaluated by chi-square or Fisher test, as appropriate, whereas the Student test was used for continuous variables. A P-value of less than 0.05 was considered as significant.

The crude odds ratios (ORs) that link HCV treatment failure to potential risk factors (age, gender, previous IFN-based treatment, HCV RNA genotype, presence of cirrhosis versus F3 fibrosis stage, bilirubin levels, platelets count, and DAA treatment regimens) were calculated by univariate analysis. Adjusted ORs were calculated by multiple logistic regression analysis to identify variables that were independently associated with failure. The reference category for OR estimates was that of the most favorable levels of exposure. HCC incidence was reported as *de novo* HCC in patients without the HCC diagnosis at baseline, prior to start of treatment.

### Ethics

The study was conducted in accordance with the guidelines of the Declaration of Helsinki and the principles of Good Clinical Practice. The study protocol was approved by the Ethic Committee of Istituto Superiore di Sanità (Italian National Institute of Public Health) and by the local Ethics Committees of each clinical center. The patients’ data were evaluated through an anonymous analysis, adopting codes generated by the electronic case-report form. Informed consent had been obtained from each patient participating in this study.

## Results

### Patient characteristics at the first DAA treatment, by genotype and DAA regimen

From January 2015 to June 2016, 3,869 consecutive patients underwent IFN-free DAA treatment. Of these patients, 3,691 (95.3%) reached the SVR12, 139 (3.6%) did not achieve the SVR12, and 39 (1.1%) did not complete the 12-week post-treatment evaluation. The baseline characteristics of the patients who completed the DAA treatment according to the response to treatment are reported in Table 1. No differences were observed between those who reached SVR12 and those who did not in terms of age, gender, liver function tests (transaminase levels) and Meld score, whereas HCV RNA genotype 3, Child Pugh class B/C, bilirubin levels higher than 1.5 mg/dl and platelets count equal or less than 120,000/mm^3^ were significantly more represented among non-responders. SOF+ribavirin (RBV) compared to the use of 2DAAs combination regimens, was more frequently used among non-responders. According to the logistic regression analysis, an independent association was found between treatment failure and older age, genotype 3 (OR 1.9; 95% CI: 1.1–3.5), bilirubin levels higher than 1.5 mg/dl (OR 1.8; 95%CI: 1.1–3.4), platelet count lower than 120,000/mm^3^ (OR 1.9; 95% CI: 1.1-3-4) and SOF+ RBV regimen (OR 2.6; 95% CI: 1.6–4.3) (Table 2).

**Table 1:** Characteristics of the study patients according to SVR following the first DAA treatment.

Variables	N. of patients Failure 139 (%)	N. of patients SVR12 3,691 (%)	P value
**Age**	58 years (range: 34–85)	59 years (range: 34–84)	0.1
**Female/Male**	31 (22.3)	993 (26.9)	0.2
**Male**	108 (77.6)	2,698 (73.1)	
**IFN experienced**	63 (45.3)	1,801 (48.7)	0.4
**Naive**	76 (54.7)	1,890 (51.2)	
**Genotypes 1**	80 (57.6)	2,622 (71.0)	<0.001
**Genotype 2**	9 (6.4)	547 (14.8)	
**Genotype 4/5**	12 (8.6)	182 (4.9)	
**Genotype 3**	38 (27.3)	340 (9.2)	
**F3 Fibrosis stage**	17 (12.2)	658 (17.8)	0.02
**F4/Cirrhosis** **Child Pugh Class A**	86 (61.2)	2945 (79,8)	
**F4/Cirrhosis** **Child Pugh Class B/C**	36 (25.8)	746 (20.2)	
**Bilirubin levels ≤1.5 mg/dl**	103 (74.1)	3,215 (87.1)	<0.001
**Bilirubin levels >1.5 mg/dl**	36 (25.8)	476 (12.9)	
**Platelets count >120,000/mm**^**3**^	63 (45.3)	2,190 (59.3)	<0.001
**Platelets count ≤120,000/ mm**^**3**^	76 (54.7)	1,501 (40.6)	
**Median Meld score (Range)**	9.7 (6–20)	9.2 (6–18)	0.3
**Median Alanine Aminotransferase Levels U/l (Range)**	66 (49–120)	57 (43–134)	0.2
**Median Aspartate Aminotransferase Levels U/l (Range)**	72 (49–102)	68 (55–98)	0.2
**2DAA±RBV treatment**	71 (51.1)	3,049 (83)	<0.001
**SOF/RBV treatment**	68 (48.9)	642 (17)	

**Table 2:** Univariate and logistic regression analysis linking failure with independent variables.

Variables	Crude OR 95% CI	Adjusted OR 95% CI
**Age**	0.97 (0.95–0.99)	0.97 (0.94–0.98)
**Female**	0	
**Male**	1.7 (0.9–2.6)	1.3 (0.8–2.2)
**IFN experienced**	0	
**Naive**	1 (0.68–1.46)	1.5 (0.9–2.2)
**Genotypes 1**	0	
**Genotype 2**		
**Genotype 4/5**		
**Genotype 3**	3.4 (2.2–5.4)	1.9 (1.1–3.5)
**F3 Fibrosis stage**	0	
**F4/Cirrhosis**	1.79 (0.9–3.4)	1.25 (0.6–2.5)
**Bilirubin levels ≤1.5**	0	
**Bilirubin levels >1.5**	2.08 (1.4–3.06)	1.8 (1.1–3.4)
**Platelets count >120,000**	0	
**Platelets count ≤120,000**	1.68 (1.2–2.4)	1.9 (1.1–3.4)
**2DAA±RBV treatment**	0	
**SOF/RBV treatment**	2.5 (1.8–3.6)	2.6 (1.6–4.3)

Failure rates following the first DAA regimen, according to HCV genotype and treatment regimen are reported in Table 3. The highest failure rate (9.6%) was observed for patients treated with SOF+RBV, for all genotypes except genotype 2, for which the failure rate was low (1.6%). The failure rate for SOF+SIM ±RBV, when used for genotypes 1 and 4, for which it was recommended, was 5.2%. Overall, the failure rate of the regimens that are no longer recommended (SOF+RBV) and those considered to be suboptimal (SOF/SIM±RBV), was 7.6% (106/1,393 patients), whereas for the regimens that are currently deemed appropriate, it was 1.4% (33/2,437 patients). In IFN-experienced patients, RBV was used and/or treatment was extended from 12 to 24 weeks, as recommended by guidelines. Only in 2 (1.4%) of the 140 patients (IFN-naïve, genotype 3, with cirrhosis), the use of SOF+DCV for 24 weeks was not associated with the use of RBV, as recommended. Lastly, a high failure rate (50–100%) was recorded in a few patients with HCV genotypes 2, 3 and 5, who received inappropriate treatment with SOF+SIM±RBV.

**Table 3:** Failure rates following the first DAA regimen, by HCV genotype and treatment regimen in patients who completed the 12 weeks post treatment evaluation (n = 3,830 patients).

	Overall	HCV genotype N. of failures/N. of treated patients (%)					
DAA regimen	N. of failures/N. of treated patients (%)	1a	1b	2	3	4	5
	139/3830 (3.6)						
**SOF+RBV**	**68/710** **(9.6)**	5/15 (33.3)	20/56 (35.7)	8/499 (1.6)	32/132 (24.2)	3/8 (37.5)	-
**SOF+SIM±RBV**	**38/683** **(5.6)**	8/99 (8)	24/520 (4.6)	1/2 (50)	1/1 (100)	3/60 (5)	1/1 (100)
**SOF+LDV±RBV**	**16/1002** **(1.6)**	3/200 (1.5)	10/752 (1.3)	-	0/1 (0)	3/44 (6.8)	0/5 (0)
**3D±RBV**	**9/894** **(1)**	3/86 (3.5)	6/806 (0.7)	-	-	0/2 0	-
**2D+RBV**	**2/64** **(3.1)**	-	-	-	-	2/59 3.4%	0/5 (0)
**SOF+DCV±RBV**	**6/471** **(1.3)**	0/47 0	1/115 (0.9)	0/55 (0)	5/244 (2)	0/10 (0)	
**SIM+DCV**	**0/6** **(0)**	-	0/6 (0)	-	-	-	-

### Second DAA regimen following failure

After a median time of 6 months (range: 5–14 months) from the first treatment failure, 72 (51.8%) of the 139 patients who failed to achieve the SVR12 were treated with a second DAA regimen, with or without RBV, with treatment lasting 24 weeks, as recommended by the most recent guidelines of the European Association for the Study of the Liver (EASL) [19]. All the 72 retreated patients had liver cirrhosis. The second DAA regimen consisted of: i) SOF/DCV in 38 patients (52.8%) (of whom 23, who had either genotype 1a or 3, also received RBV), ii) SOF/LDV in 27 patients (37.5%) (of whom 13, who had either genotype 1a or 4, received RBV), or iii) other DAA regimens in 7 patients (9.7%), as shown in Table 4.

**Table 4:** Patients who received a second DAA treatment, by type of first regimen and HCV genotype (n = 72 patients).

	Second DAA Regimen										
First DAA Regimen	SOF+DCV Number of patients (%)					SOF+LDV Number of patients (%)				Other Regimens	
	N. patients 38 (52.7%)	Gt1	Gt2	Gt3	Gt4	N. patients 27 (37.5%)	Gt1	Gt3	Gt4	N. patients 7 (9.7%)	Gt1, Gt3, Gt4
**SOF+RBV**	29	7* (24.1)	3 (10.3)	19* (65.5)		14	11 (78.6)	1(7.1)	2(14.2)	1	PEG+SOF+RBV (Gt3)
**SOF+SMV**	9	7 (77.8)	-	-	2 (22.2)	13	13* (100)			2	3D (Gt1); 2D (Gt4)
**SOF+LDV**	-	-	-	-	-	-	-	-	-	4	3D; (Gt1) SOF+SIM (Gt1 and Gt4)
**SVR12**	36 (94.7)	13 (92.8)	3 (100)	18 (94.7)	2 (100)	26 (96.3)	23 (95.8)	1 (100)	2 (100)	8 (100)	

*1 patient Gt1 and 1 patient Gt3 did not achieved the SVR12 to the retreatment with SOF+DCV; 1 patient Gt1 did not achieved the SVR12 to the retreatment with SOF+LDV

Overall, the SVR was achieved in 69 (95.8%) of the 72 patients. Among the 38 patients treated with SOF/DCV, 36 (94.7%) achieved SVR12 and 2 (5.3%) had treatment failure (one with genotype 1 and one with genotype 3). Of the 27 patients treated with SOF/LDV, 26 (96.3%) achieved SVR12, and 1 (3.7%) experienced treatment failure (genotype 1a). The few patients retreated with other regimens, as reported in Table 4, did not show failure following retreatment.

### Modifications of liver disease stage following DAA failure and retreatment

**Fig 1:**
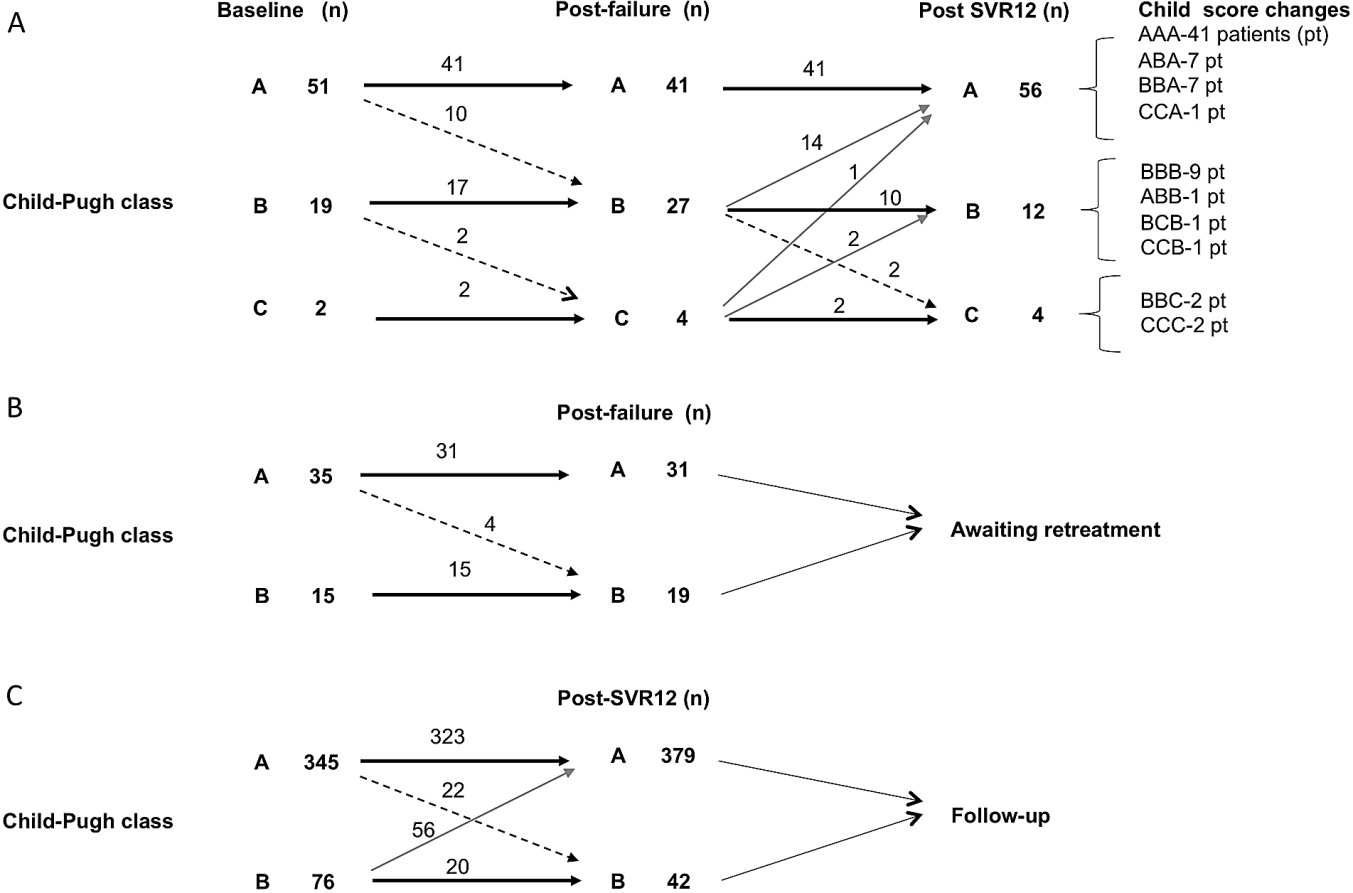
Modifications of liver disease stage following DAA treatment in patients with cirrhosis. (A) Baseline, post-failure and post-retreatment SVR12 changes of Child Pugh Class; (B) baseline and post-failure changes of Child Pugh Class for patients who were not retreated yet; (C) baseline and post-SVR12 changes of Child Pugh Class for patients who achieved SVR12 following the first DAA treatment. Bold arrows indicate patients who did not change the Child Pugh Class. Dashed arrows indicate patients who worsened the Child Pugh Class, whereas the grey arrows indicate patients who improved the Child Pugh class. In the curly brackets are reported the number of patients for specific changes observed in the Child Pugh classes in the three points of evaluation. n = number of patients.

Fig 1A shows the modifications in disease severity in the 72 patients who had cirrhosis and were retreated. Following treatment failure to retreatment (median time 6 months; range: 5–14 months), the Child-Pugh class worsened in 12 (16.7%) patients from A to B in 10 patients (19.6%) and from B to C in 2 patients (10.5%), whereas it remained unchanged in the remaining 60 patients. Following the retreatment SVR12 to the end of the study (median time of 6 months; range: 3–12 months), the Child-Pugh class improved in 17 (23.6%) patients: from B to A in 14 (19.4%) patients, from C to A in 1 patient (1.4%) and from C to B in 2 (2.9%) patients; it remained unchanged in 53 patients (73.6%) and worsened in 2 (2.8%) patients.

Following the first DAA failure, in patients with an increase in the Child-Pugh class, the mean MELD score pre-treatment was 9 (range: 7–13) and post-treatment failure was 12 (range: 9–15) (p<0.05). In patients in whom the Child-Pugh class was unmodified after failure, the mean pre- and post-treatment MELD score was 8 (range: 6–9) in Child-Pugh class A patients, while in Child-Pugh B class patients the pre-treatment MELD score was 12 (range: 9–21) and the post-treatment score was 13 (range: 9–17) (p = 0.03). Following the retreatment SVR, in Child-Pugh class B patients with a MELD score >12, the median MELD score significantly improved from 15 to 10 (P<0.001), whereas it did not change in the remaining retreated patients.

Both ascites and encephalopathy showed a numerical increase after treatment failure, followed by a decrease after SVR12, although the numbers were too small to allow for a meaningful statistical comparison (data not shown). No variceal bleeding or episodes of Spontaneous Bacterial Peritonitis occurred during the study period. Of the 72 patients who were retreated, 3 (4%) had undergone OLT before retreatment, for liver failure and/or they had developed HCC (all reached SVR12 following retreatment) and 2 (2.8%) underwent OLT after having achieved retreatment SVR12 (one in Child-Pugh class B cirrhosis and one for HCC development). Two (70%) of the 3 patients who also failed to achieve SVR12 after retreatment, and 2 (2.8%) of the 69 patients who achieved retreatment SVR12 died from liver failure complications (Child-Pugh class deteriorated from B to C) or from HCC.

Of 67 (4%) patients who were awaiting retreatment following the study period (median time of follow-up 6 months: range 5.3–12.4 months), 50 had liver cirrhosis, 35 in Child Pugh A class and 15 in Child Pugh B class. Of 35 patients in Child Pugh A class 4 (11.4%) worsened in Child Pugh B class during the study period whereas the remaining did not change it (Fig 1B). Pre-treatment Meld score was 8 (range: 6–17) whereas during the awaiting treatment follow up period (until the end of the study) it was 9.2 (range: 7–17) (p = 0.2).

Of patients who achieved the SVR following the first line DAA therapy an interim analysis of short time clinical outcomes was available for 481 patients with liver cirrhosis who had at least more than 3-month follow-up (median follow-up 5.5 months: range 3.8–9.4 months) following SVR. Among them, in the 76 Child-Pugh class B patients the classification improved to Child-Pugh A in 56 patients (74%), while in 345 Child-Pugh class A patients, the classification remained stable in 323 patients (94%) and worsened to Child-Pugh B in 22 patients (6%) (Fig 1C).

### HCC incidence

Following treatment failure, *de novo* HCC was observed in 16 (13.8%) of the 116 patients with cirrhosis, free of HCC at baseline (6 of 122 patients with liver cirrhosis in whom HCC was present prior to the start of first DAA treatment were excluded by this analysis). In particular, it was diagnosed at the end of treatment (or 1 month after the end of treatment) in 6 patients (37.5%) and during a median follow-up of 6 months (range: 3–11 months) in 10 patients (62.5%). At the retreatment SVR12, HCC was diagnosed in 4 of the 56 patients (7.1%) free of HCC at start of the retreatment.

## Discussion

The overall failure rate for DAA in a large, consecutive series of HCV patients with advanced liver disease in this multicenter, real-life study was 3.6%, which is similar to or even lower than rates reported by clinical trials [20,21,22]. The low failure rate was achieved despite the fact that regimens containing SOF+RBV and SOF/SIM, which are no longer recommended, were used in 1,393 patients (36.4%), given that they were the only regimens available at the time. The results of this study indicate that SOF/RBV treatment, genotype 3, and the severity of liver disease (as indicated by high bilirubin levels and low platelet counts) are significantly associated to failure. The length of DAA treatment and the use of ribavirin for each patient were applied in accordance with European Association for the Study of the Liver recommendations, which could be one of the main reasons for the low relapse rate, despite the use of regimens that are no longer recommended or suboptimal in this subset of difficult to treat patients.

Although sofosbuvir is a highly potent inhibitor of the NS5B polymerase, with a high potency and a high barrier to resistance, its use as DAA monotherapy in combination with ribavirin is currently not recommended [20–22]. This treatment regimen was mainly used until mid-2015 because it was the only option available for patients infected with HCV genotype 2 or 3 [16]. The results of our study strongly confirm the high failure rate following the use sofosbuvir as DAA monotherapy in all but HCV genotype 2 infected patients. The low failure rate observed in this study in patients infected by HCV genotype 2 was possibly because of the treatment duration (16–24 weeks in most of the patients). In accordance with the scientific guidelines of the American Association for the Study of Liver Diseases, the European Association for the Study of the Liver and the Asian Pacific Association for the Study of the Liver the use of at least 2 DAAs has significantly decreased the failure rate in all genotypes, as clearly indicated by the results of this study [20–22]. SOF/SIM was the first regimen available following SOF/RBV, which explains its wide use in this population. Our data confirm that the failure rate of SOF/SIM was significantly lower than SOF/RBV for genotypes 1 and 4. Interestingly, SOF/SIM showed a failure rate similar to that for SOF/LDV when used for genotype 4, whereas this did not hold true for genotype 1 (SOF/SIM vs. SOF/LDV vs. SOF/DCV: 5.2% vs 1.4% vs 0.6%, respectively; P<0.001). In fact, we observed that when new DAA regimens became available, the failure rate dramatically decreased, from 7.6% (failure rate observed in the first period of treatment, 2015) to 1.4% in the second period (end of 2015–2016). These findings are particularly relevant given the fact that in Italy access to DAA treatment was initially restricted to patients with a clinical or elastography diagnosis of liver cirrhosis and to patients with advanced liver fibrosis.

Regarding retreatment with a second DAA regimen, all patients who failed to the first DAA treatment were eligible to retreatment. Most of these patients (106 of 139: 77%) were previously treated with SOF+RBV or SOF/SIM. During the study period, 72 (67.3%) of them received a second treatment by adding a second DAA (when SOF+RBV was used in the first treatment) or a regimen containing an NS5A inhibitor, in accordance with the European Association for the Study of the Liver guidelines. Of the remaining 67 not retreated patients, 38 (35.8%) treated with a first line SOF/RBV or SOF/SIM (30 in Child Pugh Class A and 5 in Child Pugh B class) were awaiting second treatment access (median time from failure to the end of the study 5 months: range 2–8 months) whereas 29 (87.9%) treated with a first line NS5A inhibitor were waiting for the resistance test or more appropriate future DAA regimens. Considering the presence of advanced fibrosis in most of the patients, prompt re-treatment to halt viral replication and disease progression would be more than desirable. Thus to guarantee the most appropriate therapeutic option considering the only available regimens reported in this study, a resistance test could be performed to minimize the risk of a second treatment failure.

The most important results of this study deal with the clinical events for who first failed to eradicate HCV, from baseline to treatment outcome, which were prospectively monitored both after the first DAA course and following retreatment. In this regard, we hypothesized that the deterioration of the Child-Pugh class as well as the first appearance of signs of liver decompensation, such as ascites and/or encephalopathy, and liver related deaths could be associated with continuous HCV replication and therefore liver necro-inflammatory activity [23–25]. As previously reported, improvement in important clinical outcomes has been observed in cirrhotic patients following SVR [7,26]. In our study, following SVR12 after retreatment, an improvement in Child-Pugh class was observed in 17 (23.6%) of the patients with decompensated liver disease whereas in part of patients for whom the Child-Pugh class did not change (i.e., class A patients) and electrography data were available, liver stiffness significantly improved following successful retreatment (data not shown).

The results of this ongoing study on patients who obtained the SVR12 in PITER cohort, further confirms the benefit of obtaining SVR in clinical outcomes even in patients who had cirrhosis.

However, as previously reported in patients with HCV-induced cirrhosis and SVR, although there is a reduction in risk in patients with cirrhosis, particularly in those with advanced liver cirrhosis, the progression of liver disease may continue regardless of viral eradication [27–33]. Our data confirm these findings, showing that in a small proportion of patients, liver disease stage deteriorated, despite the patients’ having achieved SVR, and that only a slight improvement in ascites or encephalopathy was observed in some patients. Furthermore, for 5 (6.9%) of the patients who failed the first line DAA therapy, OLT was deemed necessary and 2 (2.8%) of the patients died despite having achieved viral eradication after retreatment. These findings suggest that we should proceed with caution, given that in some patients with advanced liver disease, liver function may not improve, or it may even worsen, regardless of viral eradication.

We observed *de novo* HCC incidence in 13.8% of the patients who experienced treatment failure. Several concerns have been recently raised on the potential role of antiviral therapy in the higher occurrence of HCC in DAA-treated patients with cirrhosis [25,34]. Moreover, a higher incidence of HCC was reported in patients who experienced treatment failure, compared to those who achieved SVR in the same stage of liver disease in a prospective study [35,36]. The frequency of *de novo* HCC was relatively high in this study, and a potential role of HCC in decreasing the chance of successful DAA treatment, which has been recently reported, cannot be ruled out [25,34,35,37,38]. To this regard, we should stress that the majority of HCCs (70%) were observed within 6 months of the end of treatment; it is thus reasonable that these cancers were sub-clinically present before DAA treatment.

In conclusion, the results of this study, which are based on data collected in a real-life setting, suggest that the failure rate following the first DAA regimen is similar to or lower than the rates reported in clinical trials. This finding is particularly noteworthy given the fact that the majority of patients who failed to achieve the SVR12 were treated with DAA regimens that are now considered as inappropriate taking into account also the severity of liver disease of the treated population. Although treatment failure is uncommon, its clinical consequences are important. Interim results showed that patients who experienced treatment failure had a worsening of liver disease, which was nevertheless “rescued” by successful retreatment in the majority of cases. This notwithstanding, some patients had no improvement in liver function following successful retreatment, which suggests that some caution should be taken in evaluating the potential clinical outcome of viral eradication in patients with advanced liver disease. Thus it must be stressed that “curing” HCV might not be the ultimate goal in patients with severe liver disease, although this study was not specifically designed to identify patients who do not benefit from viral clearance.
